# Cooperative activation of *Xenopus* rhodopsin transcription by paired-like transcription factors

**DOI:** 10.1186/1471-2199-15-4

**Published:** 2014-02-06

**Authors:** Sarah E Reks, Vera McIlvain, Xinming Zhuo, Barry E Knox

**Affiliations:** 1 Departments of Neuroscience & Physiology, Ophthalmology and Biochemistry & Molecular Biology, State University of New York Upstate Medical University, Syracuse, NY 13210, USA

## Abstract

**Background:**

In vertebrates, rod photoreceptor-specific gene expression is regulated by the large Maf and Pax-like transcription factors, Nrl/LNrl and Crx/Otx5. The ubiquitous occurrence of their target DNA binding sites throughout rod-specific gene promoters suggests that multiple transcription factor interactions within the promoter are functionally important. Cooperative action by these transcription factors activates rod-specific genes such as rhodopsin. However, a quantitative mechanistic explanation of transcriptional rate determinants is lacking.

**Results:**

We investigated the contributions of various paired-like transcription factors and their cognate cis-elements to rhodopsin gene activation using cultured cells to quantify activity. The *Xenopus* rhodopsin promoter (*XOP*) has a bipartite structure, with ~200 bp proximal to the start site (RPP) coordinating cooperative activation by Nrl/LNrl-Crx/Otx5 and the adjacent 5300 bp upstream sequence increasing the overall expression level. The synergistic activation by Nrl/LNrl-Crx/Otx5 also occurred when *XOP* was stably integrated into the genome. We determined that Crx/Otx5 synergistically activated transcription independently and additively through the two Pax-like cis-elements, BAT1 and Ret4, but not through Ret1. Other Pax-like family members, Rax1 and Rax2, do not synergistically activate XOP transcription with Nrl/LNrl and/or Crx/Otx5; rather they act as co-activators via the Ret1 cis-element.

**Conclusions:**

We have provided a quantitative model of cooperative transcriptional activation of the rhodopsin promoter through interaction of Crx/Otx5 with Nrl/LNrl at two paired-like cis-elements proximal to the NRE and TATA binding site. Further, we have shown that Rax genes act in cooperation with Crx/Otx5 with Nrl/LNrl as co-activators of rhodopsin transcription.

## Background

**Figure 1:**
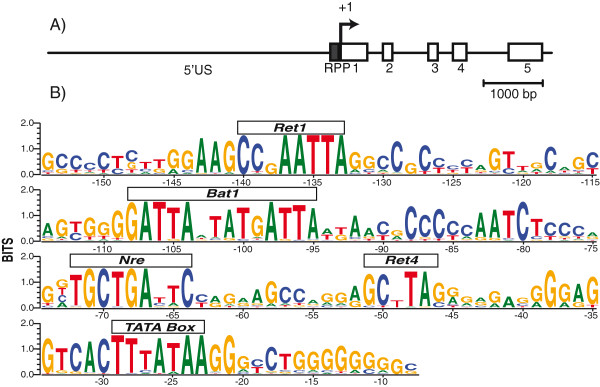
**Highly conserved vertebrate RPP. (A)** A schematic representation of the rhodopsin gene, which consists of 5′ upstream sequence (5′ US), the proximal promoter (RPP, black box), the transcription initiation site (+1) and 5 exons (white boxes). **(B)** Sequence logo representation of an alignment of 33 vertebrate proximal promoters. Approximately 200 bp around the NRE were aligned using CLUSTALW and logos created using WEBLogo3.0. The species used included seven primates, five rodents, one erinaceid, two lagomorphs, three ungulates, one camelid, one loxodonta, two carnivores, one cetacean, one tenrec, one megabat, two marsupials, one monotreme, one reptile, two birds and two *Xenopus*. Rhodopsin promoters from fish did not fit this alignment.

Rhodopsin is an abundantly expressed gene specifically found in rods, and its expression is primarily regulated at the level of transcription (for a recent review see [1]). The principal transcriptional control sequences, called the rhodopsin proximal promoter (RPP), reside within ~200 bp proximal to the transcription start site [2,3]. Additional regulatory sequences in mammalian genes are upstream of the RPP [4,5]. Alignment of 33 tetrapod RPP sequences reveals an extraordinarily high degree of conservation not observed with fish or invertebrates (Figure 1), with four conserved cis-elements (Ret1, BAT1, Ret4 and NRE) immediately upstream of the TATA box. NRE, a Maf family recognition element (MARE) site forty nucleotides upstream of the TATA box, is a target for Nrl (neural retina-specific leucine zipper protein), a transcription factor expressed exclusively in rods [6]. Flanking the NRE are three highly conserved paired-like homeodomain binding sites, Ret4, BAT1 and Ret1. These are target sites for several paired-like homeodomain transcription factors, most notably Crx but also Otx2 and Rax [7-11] Crx (cone rod homeobox protein) is expressed in both rods and cones. Nrl and Crx are highly conserved among vertebrates and are essential for rhodopsin expression. Nrl knockout mice have no rhodopsin transcripts and in fact, are missing most rod-specific genes [12,13]. Crx knockout mice have greatly reduced rhodopsin expression [14]. In both knockout mice, rod differentiation is also severely affected, indicating that these genes play multiple roles in development and photoreceptor maintenance. The importance of Nrl for rhodopsin transcription is further demonstrated in transgenic *Xenopus* rods harboring NRE mutant promoters, which have drastically reduced expression of a GFP reporter gene [15]. By contrast, individual deletion of the conserved paired-like homeobox sites does not have a major effect on reporter gene expression. Nevertheless, a mechanistic basis for the combined transcriptional activity of Nrl and Crx (and their modulators) is yet to be established.

*In vitro* studies have largely supported those in transgenic animals, namely that Nrl and Crx are the key regulators of rhodopsin transcription [1]. Nrl stimulates transcription of the rhodopsin promoter via NRE in transfected non-retinal cells [15,16]. Furthermore, mutagenesis of Nrl has revealed multiple domains required for rhodopsin promoter transactivation [17,18]. Similarly, Crx alone stimulates transcription of the rhodopsin promoter in transfected non-retinal cells [7]. Although it has a more promiscuous target, Crx binds to a core sequence, TAAT [7,19]. Rax, paired-like homeobox transcription factors, bind to the Ret1 and BAT1 sites [8,20,21] and alone weakly activates the rhodopsin promoter [8,22]. Crx and Nrl interact physically and functionally *in vitro*. Crx binds to Nrl through its homeodomain [23]. The binding site on Nrl is not as well defined [17,18]. Both Crx and Nrl interact with other transcription factors, such as TBP [17], Fiz1 [24] and Qrx [21], as well as chromatin modifiers [25]. However, the most intriguing feature of rhodopsin transcriptional activation *in vitro* is its cooperative (or synergistic) activation by Crx and Nrl [15,23,26,27]. Together they transactivate the rhodopsin promoter an order of magnitude or more than the additive effect of the individual transcription factors. This has led to the hypothesis that in rods rhodopsin transcription is activated by the cooperative activity of Crx and Nrl.

Current models for transcriptional activation describe the complex interplay between distal and proximal promoter regulatory elements, transcription factors and the basal transcription machinery (for example [28-30]). The usual hypothesis postulates that expression levels are determined by accessibility of the cis-elements (regulated by chromatin environment [31]) and the availability of functionally active transcription factors [28]. This leads to the localization of transcription factors on the promoter for a significant length of time and thus promotes interaction with other general transcription factors assembling on the promoter [32]. Thus, cis-elements are thought to increase the local concentration of transcription factors and to promote protein-protein interactions. Therefore, to account for rhodopsin transcription levels, it is important to quantitatively examine the contribution of DNA binding site architecture to activation of the rhodopsin promoter by Crx and Nrl. Our aim was to characterize the arrangement of DNA binding motifs in the RPP, i.e.to determine the requirements for cooperative (synergistic) activation by Nrl-Crx. Employing an *in vitro* system coupled with mutational analysis of the RPP, we characterized the specific role of the paired-like cis-elements in activation of the *Xenopus* rhodopsin promoter by Crx and its *Xenopus* homolog, Otx5, Rax and a novel *Xenopus* Rax family member, Rax2b [20,33].

## Results

### Rhodopsin proximal promoter activity in transfected HEK293 cells

**Figure 2:**
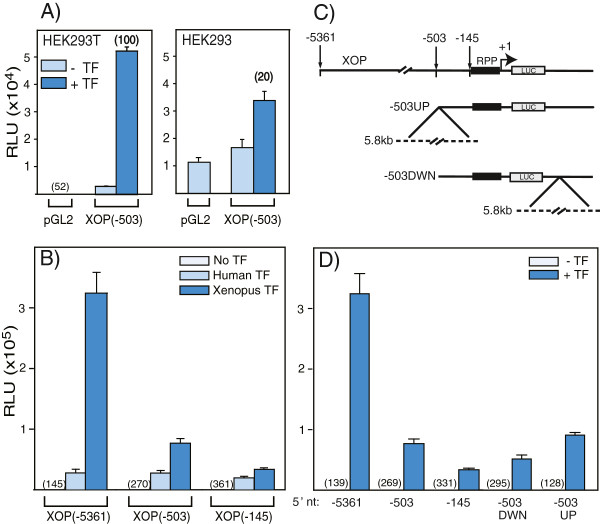
**Effect of 5′ flanking sequences on *Xenopus *rhodopsin promoter activity in transfected 293 cells. (A)** Comparison of luciferase activity (relative light units, RLU) from lysates of HEK293 or HEK293T cells transfected with a promoter-less pGL2 or XOP(−503/+41) containing luciferase plasmid in the absence (−TF) or presence (+TF) of pCS2-hCrx and pCS2-hNrl (+TF). The fold stimulation of XOP by hCrx-hNrl is indicated in parentheses. Data are mean ± S.E.M. (n = 2-6) Promoter basal activity (RLU) is shown in parentheses. **(B)** Comparison of luciferase activity in 293 T cell lysates transfected with plasmids containing different lengths of XOP 5′ US sequence: -5361, -503 and −145 all having the same 3′ sequence at +41. Cells were co-transfected with either human hCrx-hNrl or *Xenopus* LNrl-Otx5. Data are presented as mean RLU ± S.E.M. (n = 2-6). Promoter basal activity (RLU) is shown in parentheses. **(C)** Schematic of reporter constructs with additional *Xenopus* genomic DNA to control for the effect of plasmid size. All plasmids included the rhodopsin proximal promoter (RPP) upstream of the luciferase gene (LUC). XOP (−5361) contained 5.3 kb 5′ US sequence, -503UP and -503DWN contained a 5.8 kb fragment of the rhodopsin structural gene 5′ or 3′ to the RPP, respectively. The dotted line represents the inserted 5.8 kb rhodopsin gene. **(D)** Comparison of luciferase activity directed by different XOP constructs (−5361/+41, -503/+41, -145/+41, -503UP, -503DWN) in the absence (−TF) or presence (+TF) of Otx5 and L-Nrl. Activities are presented in RLU. The solid line represents the activity of samples transfected with empty pGL2 vector alone. The dotted line is a reference line for XOP(−503) to aid comparison. Data are presented as mean ± S.E.M. (n = 2-12). Promoter basal activity (RLU) is shown in parentheses.

HEK cells (human embryonic kidney cells transformed by adenovirus 5) are a versatile and rapid system in which to characterize rhodopsin promoter activation, since these cells do not express Nrl, Crx or the endogenous (human) rhodopsin gene. We compared two common lines, HEK293 (ATCC CRL-1573) and HEK293T [34,35], the latter of which expresses the SV40 large T-antigen to drive episomal replication of plasmids containing the SV40 origin of replication [36]. To quantify rhodopsin promoter activation, we transiently transfected cells with a luciferase reporter under control of various promoters and plasmids encoding Crx and/or Nrl. The activity in the absence of added transcription factors for either the promoter-less (pGL2) or a *Xenopus* rhodopsin promoter (XOP, -503/+41) containing plasmid was 1.5-fold higher in HEK293 cells compared to HEK293T cells (Figure 2A). The concentration of promoter-luciferase plasmid for all experiments was in the linear range (*data not shown*); therefore this difference in activity between the two cell lines reflects higher basal transcriptional activity independent of the specific promoter upstream of the luciferase gene.

Both cell lines exhibited robust transactivation of the XOP promoter by the combination of human Crx (hCrx) and Nrl (hNrl) or *Xenopus* homologs of Crx (Otx5) and Nrl (LNrl) (Figure 2B, [15]). However, the *Xenopus* homologs stimulated luciferase activity more strongly than their human counterparts. The higher levels of stimulation are in general agreement with our previous results, although the relative stimulation by transcription factors is somewhat higher here, mostly likely due to differences in experimental conditions.

Surprising, the activity in the presence of hNrl-hCrx was 2-fold higher in HEK293T cells compared to HEK293 cells, and contrary to the corresponding basal promoter activities (Figure 2A). It is typical to express activation by transcription factors as the relative activity, i.e. the ratio (or fold) of the activity in the presence and absence of transcription factors. For example, the activation of XOP by hNrl-hCrx is 100-fold in HEK293T compared to only 20-fold activation in HEK293 cells (Figure 2A). Thus, there is a 5-fold discrepancy in the estimated synergistic activation by hNrl-hCrx in HEK293T cells compared to HEK293 cells. This difference is a result of the higher non-specific activity of XOP as well as the lower maximal activation by hNrl-hCrx in HEK293 cells. For clarity and to facilitate comparisons between different experiments, we present all transcriptional activity data in relative light units (RLU).

Taken together, the low basal transcription and the higher Nrl-Crx stimulation in HEK293T cells are better for quantifying transcriptional stimulation of rhodopsin promoters. If we assume that the same phenomenon underlies the transcriptional differences between the two cell lines in basal activity and maximal stimulation. Therefore, we can eliminate differences in transfection efficiencies, plasmid copy number or rate of plasmid degradation. More likely are mechanisms which alter the luciferase template for transcription. One possibility is a difference in extrachromosomal plasmid packaging leading to alterations in plasmid conformation or episomal structure. Alternatively, there could be differences in the plasmid subcellular localization that lead to differences in transcription efficiency. Finally, the cells could have differences in transcription factors that lead to recognition of cryptic promoters in the plasmid backbone. It is possible that plasmids are differentially occupied by transcription complexes that are not readily displaced by hNrl-hCrx. These could account for the higher basal and lower stimulated activity in the HEK293 cells compared to HEK293T.

### *Xenopus* rhodopsin promoter 5′upstream sequence

The only region of extended conservation in tetrapod rhodopsin promoters is the RPP (Figure 1). Comparison of the 5′ upstream sequence (5′US) of *Xenopus* with other vertebrate rhodopsin promoters did not reveal any significant conserved regions (> ~ 10 bp). However, there are many core homeobox sites (ATTA, Additional file 1: Table S3), including several Crx binding sites, present in the 5′US that could contribute to transcriptional activation. Crx binding sites have also been identified in the 5′US of mouse rhodopsin by chromatin immunoprecipitation and chromosome confirmation capture [11,19]. In *Xenopus* transgenic frogs both −5361 and −503 genomic fragments drive rod-specific reporter gene expression [3]. To examine the potential modulation of transcriptional activity by regions upstream of the *Xenopus* RPP (xRPP, -145/-10, [3]), we compared two different 5′US sequences: (−5361/-146 and −503/-146) to the activity of the xRPP. We controlled for plasmid size by placing the rhodopsin structural gene upstream or downstream of the xRPP ((Figure 2D). Both 5′US sequences increased the synergistic stimulation of the xRPP by LNrl-Otx5 (Figure 2D). However, XOP(−5361/+41) stimulation was 10-fold while XOP(−503/+41) was 5-fold higher than that of the xRPP. Plasmid size does not account for this difference, since the activity of the -503UP and -503DWN plasmids, which are similar in size to the −5361 plasmid, was closer to the −503 plasmid than the −5361 plasmid (Figure 2D). The basal activity decreased 2.4-fold with increased 5′US length. We also found that while human Nrl-Crx can strongly stimulate synergistic activation, there is no difference in the level of activity between the two promoter lengths containing upstream sequence and the xRPP as compared to the *Xenopus* homologs (Figure 2B). Taken together, these results show that the RPP plays the principal quantitative role in determining transcriptional synergy by Nrl-Crx. Furthermore, the 5′US enhancement of synergistic activation appears to be specific to *Xenopus* transcription factors. Qualitatively, the relative activity of the promoters containing these 5′US sequences (−503, -5361) is similar in transgenic *Xenopus* ([3,37] and M. Haeri, *personal communication*). However, position effects and copy number variation prevent a direct comparison between transgenics and *in vitro* experiments.

### Stable integration of *Xenopus* rhodopsin promoter

**Figure 3:**
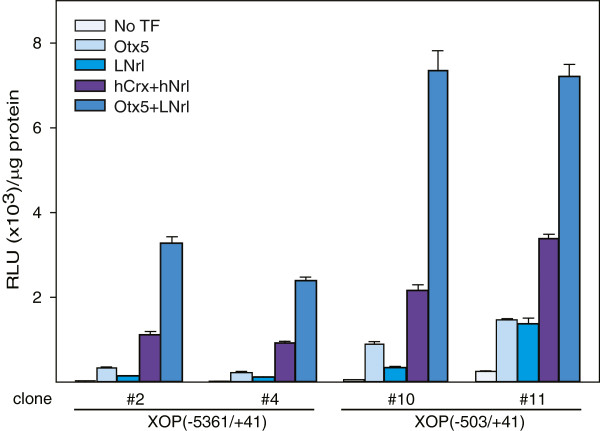
**Comparison of transcriptional activity of *Xenopus *rhodopsin promoters stably integrated into the genome of 293 cells.** Stable cell lines with integrated XOP (−5361/+41)-luciferase (#2 and #4) or XOP (−503/+41)-luciferase (#10 and #11) DNA were transfected in the absence or presence of Otx5, LNrl, Otx5-LNrl or hCrx-hNrl, and assayed for luciferase activity. Activities are presented in RLU normalized to total protein (μg) and represent mean ± S.E.M. (n = 4).

We examined whether synergistic activation of rhodopsin promoters by Nrl-Crx would occur if the promoters were integrated into the host genome. We utilized the Flp-In system, which allows for the expression of a single copy of the XOP controlled luciferase gene per cell inserted into a specific location in the genome. We generated individual clones which were hygromycin resistant, zeocin sensitive and contained XOP(−503/+41)-luciferase or XOP(−5361/+41)-luciferase. The activity in the absence of transcription factors between the four clones tested differed widely and by as much as 13 fold, with higher basal activities observed in the shorter promoter (Figure 3). LNrl individually stimulated activity with folds ranging from 5.2 to 6.2 as compared to 4.4 fold for XOP(−503/+41) and 11 fold for XOP(−5361/+41) in transiently transfected cells. Otx5 individually stimulated activity with folds ranging from 5.7 to 15.3 as compared to 6.8 for XOP(−503/+41) and 52 fold for XOP(−5361/+41) in transiently transfected cells. The human homologs, hNrl and hCrx, stimulated individually but not as robustly (data not shown). All four stable cell lines were synergistically activated by LNrl-Otx5 with increased activity ranging from 2.4 to 6.6 fold over basal + LNrl + Otx5 activity and for hNrl-hCrx, 1.7-5.1 fold. While the synergistic activation by Nrl-Crx is higher in transiently expressing HEK293T cells, this is largely due to higher basal activity of the integrated promoters. These differences could be due to the chromatin organization of the promoter DNA in an episomal environment versus an integrated state. Nevertheless, the synergistic activation of these stably integrated promoters provides support for similar behavior in rod cells.

### Role of XOP cis-elements in Otx5 activation

**Figure 4:**
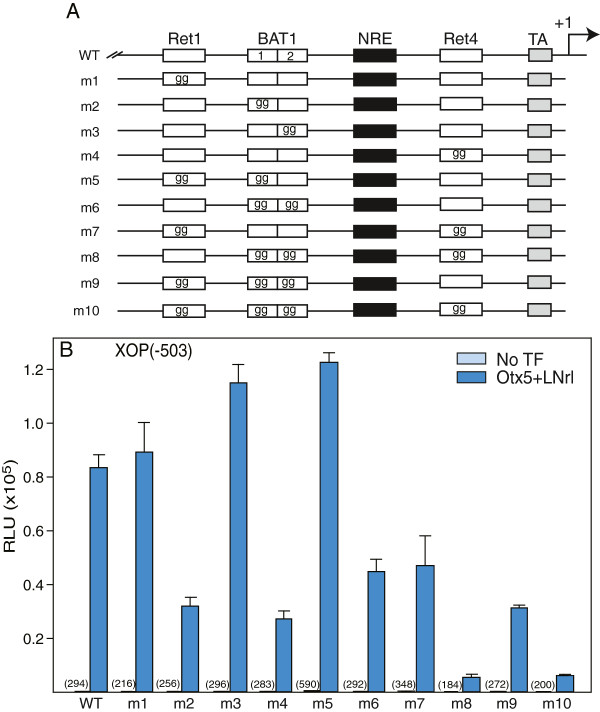
**Mutational analysis of *Pax*-like *cis*-elements in the RPP: Effect on Otx5-LNrl activation. (A)** Diagram indicating the location of the Ret1, BAT1, NRE, Ret4 and TATA box (TA) elements and the mutations (TT to GG) constructed in these sequences. The transcription start site is marked by +1. **(B)** Cells were transiently transfected with a plasmid containing a wild type promoter (XOP(−503/+41)) or one of the mutants (m1-m10) in the absence (No TF) or presence of Otx5-LNrl. Activities in cell lysates are presented as mean ± S.E.M (n = 6-8). Promoter basal activity (RLU) is shown in parentheses.

Our goal is to determine the quantitative contribution of the conserved cis-elements to synergistic activation of the rhodopsin promoter. Previous results suggested that all four conserved elements in the RPP serve a functional role in transcriptional activation. The NRE cis-element is essential for transcriptional activation of the rhodopsin promoter by Crx-Nrl [15]. Deletion analysis in HEK293T cells demonstrated that all three paired-like cis-elements, Ret1, BAT1 and Ret4, contribute to the transcriptional activation by LNrl-Otx5 (Additional file 2: Figure S1, [15]). To determine more precisely the specific site(s) of Otx5 interaction, we generated single and multiple cis-element mutants in the XOP promoter and measured Otx5-LNrl stimulated reporter activity in HEK293T cells. The conserved core sequences of Ret1 and BAT1 are ATTA and of Ret4 , CTTA (Figure 1). The core sites were changed to AGGA and CGGA, respectively, to disrupt binding of paired-like transcription factors (Figure 4A). Mutation of Ret1 (m1) in XOP(−503) increased activity 17% compared to wild type (WT) (Figure 4B). The BAT1 site has two conserved ATTA sites. Mutation of the first site (m2) decreased activity by 58%, while mutation of the second BAT1 site (m3) increased activity by 26% compared to WT (Figure 4B). Mutation of Ret4 (m4) significantly decreased activity by 68% (Figure 4B). Mutation of both the Ret1 site and the first BAT1 core site (m5) increased activity in the short promoter fragment by 35%. Mutation of both BAT1 sites (m6) decreased the activity by 50%. Mutation of both Ret1 and Ret4 (m7) decreased the activity by 45%. Mutation of the two BAT1 sites in combination with Ret4 (m8) dramatically decreased the activity by 94%. Mutation of the BAT1 sites in combination with the Ret1 site (m9) decreased the activity by 61%. Mutations in all four conserved core sites (m10) strongly inhibited Otx5-LNrl stimulated activity 92%. None of the mutants were able to inhibit the activity to basal levels. Similar results were obtained for the longer XOP promoter (−5361) containing identical combinations of mutations (Additional file 3: Figure S2). These mutational studies show that Otx5 utilizes both the BAT1 and Ret4 sites equally and additively to activate the promoter in concert with LNrl. The Ret1 site and second BAT1 site do not appear to be necessary for activation but contribute to the overall level of transcriptional activation. In addition, Otx5 does not utilize Ret1 for transactivation. Furthermore, while the cis-elements in the proximal promoter are required for maximal Otx5-LNrl activation, the 5′US contributes to XOP activation. We can conclude that Ret1 has a functional role distinct from BAT1 and Ret4 in this assay. Only one of the BAT1 AATA sites is important for transactivation despite the strong conservation of both.

### Identification of a novel Rax family member

Using a yeast one hybrid screen with the Ret1 cis-element (GCCAATTAA) as bait and a *Xenopus* laevis adult retina cDNA library, we identified a novel gene, which we name Rax2b (Accession number: JN392465). Rax2b is a member of the Rax paired-like homeodomain transcription factor family and in sequence comparisons is the paralog of Rax2a [20] (89.9% amino acid). Rax2b was detected by RT-PCR in embryos at stage 35/36 (Additional file 4: Figure S3A). All Rax family genes were expressed in the adult retina with Rax1a the most highly expressed followed by Rax2b, Rax2a and Rax1b (Additional file 4: Figure S3B).

### Co-activation of rhodopsin transcription by the Rax family

**Figure 5:**
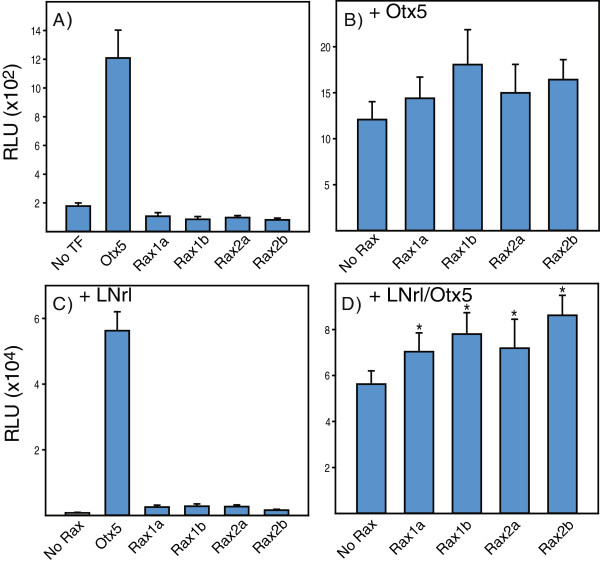
***Xenopus *Rax family transcription factors co-activate the rhodopsin promoter with Otx5-LNrl.** Comparison of luciferase activity in lysates from cells transfected with XOP(−503/+41) alone or individually with **(A)** Otx5, Rax1a, Rax1b, Rax2a, Rax2b; **(B)** Otx5 in combination with Rax1a, Rax1b, Rax2a or Rax2b; **(C)** LNrl in combination with Rax1a, Rax1b, Rax2a or Rax2b; **(D)** LNrl/Otx5 in combination with Rax1a, Rax1b, Rax2a or Rax2b. All activities are presented as mean RLU ± S.E.M. (n = 6).

**Figure 6:**
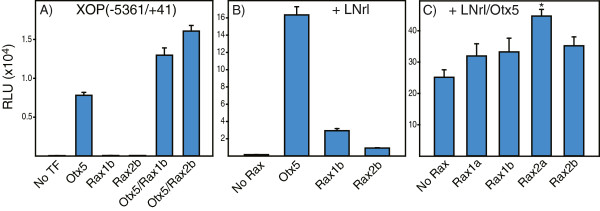
**Sequences upstream of the rhodopsin RPP enhance co-activation by *Xenopus *Rax family transcription factors.** Comparison of luciferase activity in lysates from cells transfected with XOP(−5361/+41) alone or individually with **(A)** Otx5, Rax1b, Rax2b or in combination; **(B)** LNrl in combination with Otx5, Rax1b or Rax2b; **(C)** LNrl-Otx5 in combination with Rax1a, Rax1b, Rax2a or Rax2b. All activities are presented as mean RLU ± S.E.M (n = 6).

We measured the activity of two rhodopsin promoter fragments (−5361/+41, -503/+41) co-expressed with individual members of the Rax family alone or in combination with LNrl and/or Otx5 in transfected HEK293T cells. Individually, each Rax transcription factor decreased basal activity of XOP(−503/+41). In combination with Otx5 or LNrl, Rax did not significantly increase activity (Figure 5A-C). All four Rax transcription factors in combination with Otx5-LNrl increased activity (Figure 5D) by 17% (Rax1a), 26% (Rax1b), 20% (Rax2a) and 31% (Rax2b). Similar results were observed for the longer promoter (−5361/+41) [22% (Rax1a), 23% (Rax1b), 45% (Rax2a) and 29% (Rax2b)] (Figure 6A-C). These data demonstrate that Rax family TFs are co-activators with Nrl and Crx of rhodopsin transcription.

### Role of xRPP cis-elements in Rax co-activation

**Figure 7:**
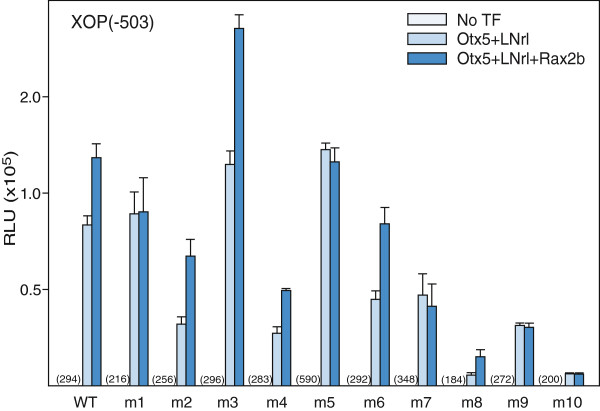
**Mutational analysis of *Pax*-like *cis*-elements in the RPP: Effect on Rax co-activation.** Comparison of luciferase activity in lysates from cells transfected with XOP(−503/+41) or RPP mutants *m1-m10*alone, with Otx5-LNrl or with Otx5-LNrl-Rax2b. Activities are presented as mean RLU ± S.E.M. (n = 6-8). Promoter basal activity (RLU) is shown in parentheses.

To determine the xRPP cis-element(s) utilized by Rax, we measured Otx5-LNrl-Rax2b stimulated promoter activity in the deletion and the XOP promoter mutants in transfected HEK293T cells. Rax2b increased Otx5-LNrl stimulated activity by 30% in the BAT1Δ mutant but had no effect on the level of activity of the Ret1Δ or Ret1/BAT1Δ mutants (Additional file 5: Figure S4). Rax2b co-activation was abolished in the XOP promoters with a mutated Ret1 site (net decrease: m1(32%), m5 (35%), m7(22%), m9 (13%), m10 (killed) but preserved in the promoters containing mutations in the BAT1 or the Ret4 sites (net increase: m2 (22%), m3 (12%), m6 (23%), m8 (6%)) (Figure 7, Additional file 6: Figure S5). These mutant data suggest that Rax2b co-activates transcription specifically through the Ret1 site.

### Rax co-activates preferentially through the Ret1 site

**Figure 8:**
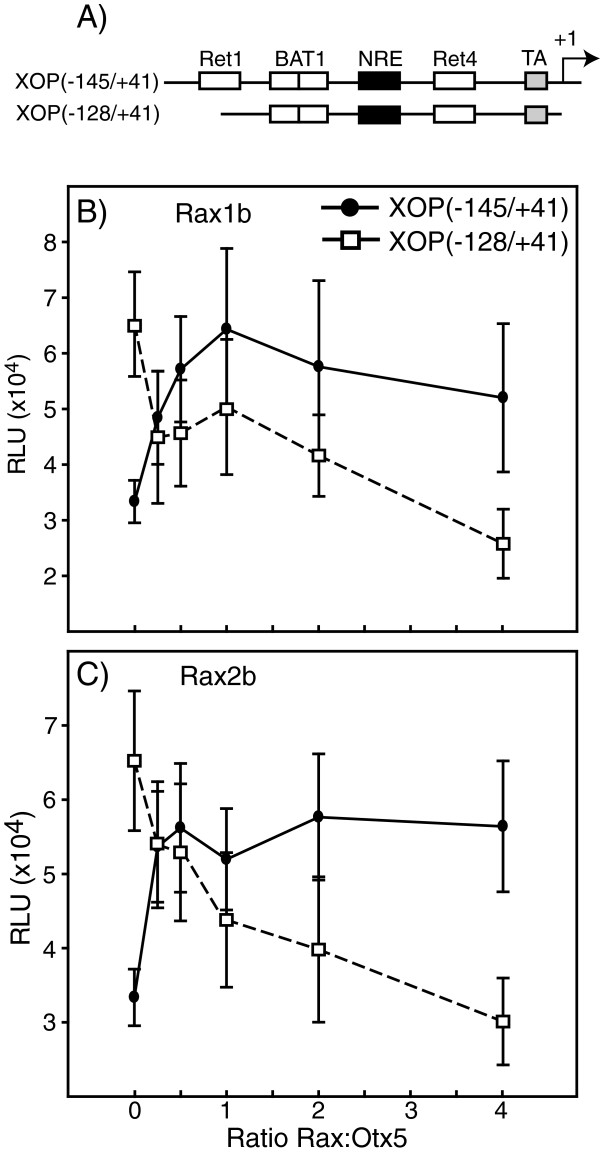
**Effect of Rax concentration on co-activation of the RPP. (A)** Schematic diagram of the plasmids containing RPP (XOP(−145/+41) or RPP missing Ret1 (XOP(−128/+41). **(B, C)** Cells were transiently transfected with either XOP(−145/+41) or XOP(−128/+41), LNrl-Otx5 and various concentrations of Rax1b **(B)** or Rax2b **(C)**. Activities are presented as mean RLU ± S.E.M. (n = 6).

To examine further the apparent preference of Rax for the Ret1 cis-element, we compared Otx5-LNrl stimulated activity with titrated concentrations of Rax1b or Rax2b using XOP (−145/+41) and a XOP fragment truncated just before the Ret1 site (−128/+41) (Figure 8A). Rax1b increased the Otx5-LNrl stimulated activity of XOP(−145/+41) maximally by 3-fold at a 1:1 ratio, plateauing at higher concentrations (Figure 8B). The Rax2b titration results were comparable (Figure 8C). These data suggest that Rax does not compete with Otx5 for the same cis-element but preferentially selects the Ret1 site for co-activation. Interestingly, a similar result was obtained titrating Otx5 in the presence of LNrl and measuring XOP(−503/+41) activity (data not shown), suggesting that Otx5 does not utilize the Ret1 site for activation. Addition of Rax1b decreased the Otx5-LNrl stimulated activity of the truncated XOP fragment (−128/+41) by 2-fold at a 0.25:1 ratio and maximally at a ratio of 4:1. Comparable results were obtained for Rax2b with an initial 1.2-fold decrease steadily dropping to below stimulatory levels of Otx5-LNrl alone. These results suggest that when the Ret1 site is abolished, Rax can compete with Otx5 for the BAT1 and/or Ret4 sites as indicated by the decrease in Otx5-LNrl stimulation. Importantly despite the ability of Rax to compete with Otx5, it cannot replace Otx5 to partner with Nrl for synergistic activation of the promoter. Thus, while Rax is able to interact with the BAT1 sight, it still preferentially selects Ret1 in the complete xRPP.

## Discussion

### Nrl-Crx synergy

**Figure 9:**
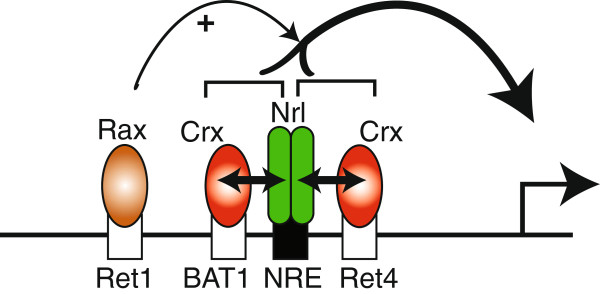
**Model of *Xenopus *rhodopsin transcriptional activation.** Nrl binds to the rhodopsin promoter via a highly conserved NRE site (TGCTGAnnC) that is immediately upstream of the TATA binding site. Crx binds independently to two adjacent sites (G/CTTA). This combination synergistically activates transcription with each Crx contributing equally to the overall activity. The Ret1 site (CCAATTA) mediates Rax protein binding to stimulate transcription.

We used HEK293T cells for quantitatively measuring transcriptional activation of the rhodopsin promoter by Nrl, Crx and Rax. The transcriptional activity of the *Xenopus* rhodopsin gene is regulated by a combination of these transcription factors interacting with multiple conserved sequence motifs. The transiently transfected cultured cells are a powerful tool for the quantitative measurement of promoter activation by individual or combinations of TFs, although they do not mimic the photoreceptor cell/native chromatin environment. We demonstrated that the *Xenopus* rhodopsin proximal promoter (RPP) is solely responsible for regulating the synergistic activation by Nrl-Crx/Otx5. These results build on previous published studies (summarized in [1]) which initially identified the essential regulatory sites in the *Xenopus* RPP: NRE, Ret1, BAT1 and Ret4 [3,15]. It has been clearly established that the NRE cis-element, which binds Nrl, can modestly activate transcription, alone, and is required for synergistic activation of rhodopsin transcription in concert with Crx/Otx5 [3,6,7,15,23]. To date, little is known about the contribution of the highly conserved paired-like cis-elements, Ret1, BAT1, and Ret4, to synergistic activation by Nrl-Crx/Otx5. Otx5 activated through the BAT1 and Ret4 sites, and both were utilized equally for synergistic activation in concert with Nrl. The Ret1 site was not utilized by Otx5 and thus, did not contribute to the synergistic activation. Based on these results and those of previous studies [21,23,38], these data support a model of rhodopsin transcriptional regulation by Nrl and Crx/Otx5 (Figure 9). An Nrl site and a Crx site within 40 bp of each other are found in many photoreceptor genes and constructs containing these cis-regulatory elements were capable of driving high levels of expression in mouse retina explants [10]. Interestingly, synthetic promoters, which contained different arrangements of Nrl/Crx motifs but lacked overall sequence homology, where able to drive robust expression in mouse retinal explants. Many photoreceptor genes are regulated by Nrl and Crx and synergistic activation has been observed often [3,7,11,13,15,25-27,39]. Thus, the arrangement and spacing of these promoter cis-elements appears to be important for generating high levels of gene expression.

A recent study quantifying bovine rhodopsin promoter activity in mouse retinal explants reported that the BAT1 site was not only essential (required) for promoter activation but was the most efficient site in driving activity [40]. The Ret4 site appeared to be less important in transcriptional activation in this live mouse cell system. Substitution of the mouse Ret1 and Ret4 sites with a BAT1 consensus sequence motif increased activity by 10-fold. Replacing the Ret4 sequence with BAT1 consensus sequence increased activity approximately 4-fold. These differences suggest that there may be species dependent differences in opsin gene regulation, either from subtle sequence variation or transcription factor complexes functional across different cells.

### Rax is a co-activator of rhodopsin

We identified a novel retinal homeobox transcription factor, Rax2b, which is a paralog of Rax1a [20]. Rax2 belongs to the aristaless-related paired-like homeobox gene family, which includes Rax1a and Rax1b. In promoter activity assays employing mutant promoter constructs, we demonstrated that while the Ret1 cis-element is not utilized by Otx5 for cooperative activation by Nrl-Otx5, it is utilized by Rax to enhance the level of synergistic activation. Furthermore, Rax activates, selectively and preferentially, through Ret1 and cannot substitute for Otx5 as a co-activator with Nrl of synergistic activation. These results suggest that the Ret1 site is used by Rax to fine tune synergistic activation by Nrl-Otx5. Based upon our results, we suggest that Rax provides the cell with an additional means of increasing rhodopsin expression levels. An unidentified repressor might also modulate transcription through the Ret1 site to inhibit transcription. Rax has been shown to bind to Ret1, BAT1 and Ret4 motifs in *in vitro* binding assays and to Ret1 by ChIP [8,20,21,41]. Previously published studies have also reported that Rax, alone, can weakly activate rhodopsin transcription and in combination with Nrl-Crx, can enhance synergistic activation [8,20,41]. The Rax family (Rax1a and b, Rax2a and b), which is found in most vertebrate species, has been implicated in eye formation, playing a critical role in cell-fate determination and development [33,41,42]. Rax1 and Rax2 continue to be expressed in the adult retina. However, the functional role of Rax in the mature retina is not clear. Qrx, a paired-like homeodomain TF, appears to be the mammalian ortholog to Rax2 [21]. It is expressed in both the outer and inner nuclear layers of the retina. In transient tranfection reporter activity assays, it activated rhodopsin expression through the Ret1 site, was a weak activator alone but enhanced Nrl-Crx synergy. Interestingly, mice do not appear to carry the Rax2 gene. However, the regulatory machinery for Rax2 seems to be intact, since transgenic mice strongly expressed an EGFP-tagged Qrx promoter fragment in photoreceptors.

### Role of rhodopsin 5′ US

The transcriptional activity assays performed on different lengths of the *Xenopus* rhodopsin promoter provide additional insights into promoter activation. While the RPP was responsible for the cooperative synergistic activation by Nrl, Crx and Rax, the 5′ upstream promoter sequence (5′ US) distal to the RPP contributed to the activation as well. The level of activity was dependent on the length of the promoter as well as the species of TFs used to activate. This observation was reinforced by the promoter activity assay data in which all three paired-like cis-elements were mutated (m6), resulting in a dramatic but not complete loss of activation. These results suggest that there are regulatory elements upstream of the *Xenopus* RPP that enhance transcription and are species-specific. This seems plausible since a distal regulatory element, the rhodopsin enhancer element (RER), which activates transcription, has been described for the mammalian promoter and is required for maximal activation of expression *in vivo*[4,5]. Although we could not identify any conserved sequence element similar to the mammalian RER, there are multiple core ATTA/CTTA sites present in the 5′US, including core Ret1 (CAATTA), BAT1 (GGATTA), Ret4 (GCTTA) and NRE (TGCTGA) motifs which could potentially bind Crx, Rax, other paired-like TFs or bZIP TFs, such as Nrl, which might contribute to the increased activity.

## Conclusions

In the present study, we characterized regulatory regions of the *Xenopus* rhodopsin promoter and their contribution to transcriptional activation. The RPP is solely responsible for synergistic activation, however, the 5′US contributes to overall transcriptional activity. We defined quantitatively the individual contributions of *Xenopus* rhodopsin promoter cis-elements to synergistic activation of transcription by the paired like transcription factors, Rax and Crx/Otx5. We showed specificity of the interaction between the transcription factors and cis-elements. Rax preferentially binds to the Ret1 cis-element and augments synergy, while Otx5/Crx binds to both the BAT1 and Ret4 cis-elements, contributing equally to synergistic activation. These studies characterizing the rhodopsin promoter architecture will advance our understanding of the role of transcriptional network machinery in rod photoreceptor development and homeostasis.

## Methods

### Vertebrate rhodopsin proximal promoter consensus sequence

Approximately 200 bp around the NRE were aligned using CLUSTALW and then manually adjusted to optimize Pax-like cis-elements. Logos were created using WEBLogo3.0. The accession numbers for the sequences used in the alignment are described in Additional file 7: Table S1 and the multiple sequence alignment is in Additional file 8.

### DNA expression constructs

pCS2-Otx5 was obtained from A. Viczian. pCS2-LNrl was subcloned from pMT-LNrl [15]. The pCS2-Rax2b expression construct was generated by releasing the full length cDNA from the yeast one hybrid vector pGADT7-Rec2 with EcoRI and XhoI and ligating into the same sites of pCS2. pCS2-Rax2a (Rx-L; NM_001095716) was generated by PCR from a cDNA clone (XL073a16) obtained from NIBB and subcloned into pCS2. pCS2-Rax1a (Rx1; NM_001088218) and pCS2-Rax1b (Rx2a; AF001049) were generated by RT-PCR from *Xenopus* laevis adult retina total RNA and subcloned into pCS2. pCS2-hNrl and pCS2-hCrx were generated by PCR from cDNA clones obtained from A. Swaroop and S. Chen, respectively. hNrl was subcloned into the BamHI and Xba I sites, and hCrx into the BamHI and Not I sites in the pCS2 vector. All constructs were verified by sequencing. See Additional file 9: Table S2 for a list of primers.

### Promoter reporter constructs

Four WT *Xenopus* rhodopsin promoter luciferase reporter constructs, pGL2-XOP (−5361/+41, -503/+41, -145/+41, -128/+41), were described previously [3]. The deletion constructs containing disrupted regions within the XOP(−503/+41) proximal promoter (Ret1Δ, BAT1Δ) were described previously [3]. The Ret1/BAT1Δ (−136/-91) deletion mutant was generated as described previously [3]. The pGL2 -503DWN plasmid was described previously [pXOP(−503/+41)luc(5800)] [3]. The pGL2 -503UP construct was generated as follows. A 5.8 kb PCR product of the *Xenopus* rhodopsin exons and 3′ region was amplified using the -503DWN plasmid as a template and cloned into the Xma I and Mlu I sites of the pGL2 basic plasmid. The XOP(−503/+41) sequence was amplified by PCR and inserted into the Nhe I and Bgl II sites just downstream of the 5.8 kb rhodopsin sequence. Mutations in the cis-elements (ATTA to AGGA) were introduced in both XOP(−5361/+41) and XOP(−503/+41) following the QuikChange XL Site Directed Mutagenesis protocol (Stratagene). The promoter constructs were verified by sequencing. The primers used in cloning are found in Additional file 9: Table S2.

### Flp-In stable cell lines

A DNA fragment that included the FRT sites and hygromycin gene was PCR amplified using the pcDNA5 FRT TOPO vector as template (Invitrogen). The purified PCR fragment was cloned into the Sal I and Bam HI sites of the pGL2-basic vector (Promega), which we named pGL2-FRT. The XOP(−503/+41) and XOP(−5361/+41) promoter fragments were then excised from their pGL2 reporter constructs with Xma I and Bam HI and cloned into the same restriction sites in the modified pGL2/FRT vector. Flp-In 293 T-REx cells (Invitrogen), containing the stably integrated FRT target sites upstream of the Zeocin gene, were co-transfected with the pOG44 plasmid, containing the Flp recombinase gene (Invitrogen), and the pGL2-FRT-XOP(−503/+41) or pGL2-FRT-XOP(−5361/+41) plasmids using Fugene 6 following the manufacturer’s protocol (Roche Diagnostics). Cells were passaged 48 h after transfection. Hygromycin B (150 μg/ml) was added to the culture medium the next day. Twelve individual stable clones expressing XOP(−503/+41)-Luciferase or XOP(−5361/+41)-Luciferase were picked 14 days after selection and expanded. Cell lines were also tested for loss of Zeocin (100 μg/ml) resistence. Only the dually selected clones were used for reporter assays. The promoter constructs were verified by sequencing. The primers used in cloning are found in Additional file 9: Table S2.

### HEK293(T) transfections

HEK293 or HEK293T cells were seeded into 24 wells at 2.4 × 10^5^ and 1.6 × 10^5^ per well, respectively. The stable Flp-In 293 T-REx cell lines were seeded at 2.4 × 10^5^. Cells were co-transfected the next day with Fugene 6 and various combinations of reporter gene and/or expression constructs at a total DNA concentration of 1 μg/well following the manufacturer’s protocol (Roche Diagnostics). After 48 h cells were harvested and lysed with 100 μl/well of Passive Lysis Buffer following manufacturer’s instructions for the Luciferase Reporter Assay System (Promega). Luciferase activity was measured in 10 μl of cell lysate using a Biotek Synergy II plate reader (Biotek Instruments, Inc.). Luciferase activity from mock transfected.

### RT-PCR

Total RNA was isolated from *Xenopus* embryo heads (stages 35/36, 39/40) or adult retinas using an RNAeasy kit (Qiagen) and analyzed using a 2100 Bioanalyzer (Agilent Technologies). Complementary DNA was synthesized from 1 μg total RNA with Superscript II RT polymerase and random hexamers (Invitrogen). cDNA was amplified over 33 cycles using Advantage 2 DNA polymerase (Clontech) and the appropriate primers for histone H4 and Rax family members (Additional file 9: Table S2).

### Real time RT-PCR

Total RNA was isolated from harvested adult *Xenopus* retinas using an RNAeasy kit (Qiagen) and analyzed using a 2100 Bioanalyzer (Agilent Technologies). Complementary DNA was synthesized from 1 μg total RNA using the Quantitect Reverse Transcription kit (Qiagen). Real time PCR reactions were comprised of 1 μl of cDNA (diluted 1:2), 5 μl of SYBR green mix (Lightcycler 480 SYBR Green I Master kit, Roche Diagnostics), 0.5 μl each of sense and antisense primers (10 pmol) to target (see Additional file 9: Table S2 for list of primers). Real time PCR was performed in a Lightcycler 480 instrument (Roche Diagnostics). The cycling parameters were 95ºC for 10 min followed by 46 cycles at 95ºC, 60ºC, 72ºC and 80ºC for 15 s, 15 s, 15 s and 5 s, respectively.

### Yeast one-hybrid assay

The MATCHMAKER one-hybrid system (Clontech) was used to isolate *Xenopus* transcription factors which might bind to a highly conserved sequence found in tetrapod L-opsin promoters, the ROP2 cis-element [43]. The bait vector was generated by ligation of an oligonucleotide containing four tandem repeats of ROP-2 (5′-GCCAATTAAGAGAT-3′) into the Xma I and Sac II sites of the pHIS2 vector. The construct was confirmed by sequencing. A cDNA library of the adult *Xenopus* retina was prepared using poly A RNA and oligo(dT) primers according to MATCHMAKER protocol except that the denaturation time during PCR was increased to 25 s. A fusion library in pGADT7-Rec(2) vector was constructed by homologous recombination in yeast. Yeast were sequentially transformed first with the bait plasmid and then the library. Transformants (6 × 10^6^) were screened on SD/-His/-Ura/-Leu supplemented with 10 mM 3-amino-1,2,4-triazole (3-AT). The screen yielded 30 primary transformants. The plasmid DNA containing the activation domain and cDNA inserts was isolated using standard techniques. The Y190 strain was transformed with these rescued library plasmids and used for mating the secondary screen. Of the 30 transformants, seven survived after mating with the original ROP2-containing Y187 strain on selective plates containing 40 mM 3-AT, but not in plates containing Y187 strain containing scrambled ROP2 sequence bait. The library cDNA inserts were rescued following standard protocols and sequenced.

## Abbreviations

TF: Transcription factor; RPP: Rhodopsin proximal promoter; TA: TATA box; XOP: *Xenopus* opsin promoter; 5′ US: 5′ upstream sequence; LNrl: *Xenopus* laevis neural retinal leucine zipper; Crx: Cone rod homeobox; Otx5: Orthodenticle-related homeobox; hNrl: Human neural retinal leucine zipper; hCrx: Human cone rod homeobox; Rax: Retina and anterior neural fold homeobox; RCOP: Red cone opsin promoter; RLU: Relative light unit; BAT1: Ret1, NRE, cis-elements in the XOP promoter.

## Competing interests

The authors declare that they have no competing interests.

## Authors’ contributions

SER carried out the molecular biology and cell transfection studies, VM performed the one-hybrid analysis and cloned Rax2b cDNA, XZ prepared the stable cell lines and contributed to their characterization, BEK carried out the sequence analysis. BEK conceived of the study. All authors participated in various aspects of the study design. SER and BEK performed the statistical analysis. The manuscript was drafted by SER and BEK, and edited by all authors. All authors read and approved the final manuscript.

## Supplementary Material

Additional file 1: Table S3 Characterization of *Xenopus* promoter sequences. Nucleotide composition and number of ATTA sites of the different *Xenopus* rhodopsin promoter lengths used in this study: XOP(−5361/+41), XOP(−503/+41) and XOP(−145/+41). Click here for file

Additional file 2: Figure S1 Effect of *cis*-element deletions on *Xenopus* rhodopsin promoter activity. Comparison of luciferase activities in lysates from cells transfected with WT XOP(−503/+41), Ret1∆(−503/+41), BAT1∆(−503/+41) or Ret1/BAT1∆ (−503/+41) alone or with LNrl-Otx5. Activities are presented as mean RLU ± S.E.M. (n = 6). Click here for file

Additional file 3: Figure S2 Mutational analysis of *Pax*-like *cis*-elements in the RPP with 5′ upstream sequences: Effect on Otx5-LNrl activation. Cells were transiently transfected with a plasmid containing a wild type promoter (XOP(−5361/+41)) or a mutation in cis-element (s) (m1-m10, *see Figure*4*A*) in the absence (No TF) or presence of Otx5-LNrl. Activities in cell lysates are presented as mean ± S.E.M (n = 6-8). Promoter basal activity (RLU) is shown in parentheses. Click here for file

Additional file 4: Figure S3 Expression of Rax transcription factors in *Xenopus laevis*. (A) RT-PCR was performed using total RNA (1 μg) from embryo heads (stages 35/36 and 39/40) or adult retinas and primers to amplify histone H4 and the three Rx paralogs (xRax1b, 2a and 2b). (B) Quantitative analysis of Rax expression levels in adult retinas by qRT-PCR. Click here for file

Additional file 5: Figure S4 Deletion analysis of *Pax*-like *cis*-elements in the RPP: Effect on Rax2b co-activation. Comparison of luciferase activities in lysates from cells transfected with WT XOP(−503/+41), Ret1∆(−503/+41), BAT1∆(−503/+41) or Ret1/BAT1∆ (−503/+41) alone or with LNrl-Otx5-Rax2b. Activities are presented as mean RLU ± S.E.M. (n = 6). Click here for file

Additional file 6: Figure S5 Mutational analysis of *Pax*-like *cis*-elements in the RPP with 5′ upstream sequences: Effect on Rax2b co-activation. Comparison of luciferase activity in lysates from cells transfected with XOP(−5361/+41) or XOP(−5361/+41) containing RPP mutants (*see Figure *4*A*) alone with Otx5-LNrl or with Otx5-LNrl-Rax2b. Activities are presented as mean RLU ± S.E.M. (n = 6-8). Promoter basal activity (RLU) is shown in parentheses. Click here for file

Additional file 7: Table S1 Accession numbers of tetrapod rhodopsin proximal promoters. Click here for file

Additional file 8 Alignment of tetrapod rhodopsin proximal promoters. Click here for file

Additional file 9: Table S2 List of primers used in this study. Click here for file
