# Supplementary material for: Cooperative activation of Xenopus rhodopsin transcription by paired-like transcription factors
Source: BMC Mol Biol. 2014 Feb 6;15:4. doi: 10.1186/1471-2199-15-4 (PMC3937059; doi:10.1186/1471-2199-15-4)
Supplement: Additional file 7: Table S1 — Accession numbers of tetrapod rhodopsin proximal promoters. [file 1471-2199-15-4-S7.pdf]

# CLUSTALW multiple sequence alignment

```
Felis           ---CCCTCTTGGAAGCCAATTAGGC--CCCCAGTTGCAGTAGTGGGGATTAGTATGATTA 55
Dolphin         ---CCCTCTTGGAAGCCAATTAGGC--CCCCAGTTGCAGCAGTGAGGATTAATATGATTA 55
Canis           ---CACTCTTGGAAGCCAATTAGGC--CCCTAGTTGCGGCAGTGGGGATTATTATGATTA 55
Chim            -GCCCCTCTTGGAAGCCAATTAGGC--CCTCAGTTTCTGCAGCGGGGATTAATATGATTA 57
Gorilla         -GCCCCTCTTGGAAGCCAATTAGGC--CCTCAGTTTCTGCAGCGGGGATTAATATGATTA 57
Orangutan       -GCCCCTCTTGGAAGCCAATTAGGC--CCTCAGTTTCTGCAGCGGGGATTAATATGATTA 57
Human           --CCCCTCTTAGAAGCCAATTAGGC--CCTCAGTTTCTGCAGCGGGGATTAATATGATTA 56
Macaque         -GCCCCTCTTGGAAGCCAATTAGGC--CCTCAGTTTCTGCAGCGGG-ATTAATATGATTA 56
Marmoset        -GGACCTCTGGGAAGCCAATTAGGC--CCTCCATTTCTGCAGCGGGGATTAATATGATTA 57
Bushbaby        GGCCCCTCTTAGAAGCCAATTAGGT--CCCCAGTAGCTGTGGTGGGGATTAATATGATTA 58
Mouse           --GCCCCTCTGCAAGCCAATTAGG---CCCCGGTGGCAGCAGT-GGGATTAGCGTTAGTA 54
Rat             -GGCCCCTCTGCAAGCTAATTAGG---CCCCAGTGGCAGCAGT-GGGATTAGTATTAGTG 55
Tree            -CGTCACCCGGGAAGCCAATTAG----CCCCGGTGACAGCGGTGGGGATTATTATGATTC 55
GuineaPig       -TGTCCCCCCACAAGCCAATTAG----GCCCTGTGGCAGTGT--GGGATTAGTATGATTA 53
Kangara         -GGCGCTTCTGCAAGCCAATTAGGCC-CCCCGGCAGCAGCGGTGGAGATTAGTATGATTA 58
Hedgehog        -GGCCCCTCTGGGCACTAATTAGG---CCCCAGCAGCCCT----GGGATTACCGCAATCA 52
Pika            -GCCCCTCTCAGAAGCCAATTAGGCC-CCGCACTTGCTACCATGGGGATTAATATGATTA 58
Rabbit          -GCCCCTTTTGGAAGCCAATTAGGCC-CC-TAGTTGCAGCCATGGGGATTAATATGATTA 57
Horse           GGCCCCTCACGGAAGCCAATTAGG---CCCCAGTTACAGCAGCGGTGATTAATATGATTA 57
Elephant        GGCCCCTCTTGGAAGCCAATTAGG---TCCCAGTTGCAA--GTGGTGATTAGTATGATTA 55
Pig             -GCCCCTCTTGGAAGCCAATTAGG---CCCCAGTTGCAGCCGTGGGGATTAATATGATTA 56
Bos             --CCCCACCTGGAAGCCAATTAAGC--CCCTCGTTGCAGCAGTGAGGATTAATATGATTA 56
Alpaca          --CCCCTCCTGGAAGCCAATTAGGC--CCCTGGTTGCGGCAGTGGGGATTAATATGATTA 56
Tenre           -GCCACTGTTGGAAGCCAATTAGC---CTTTGGTTGCAG--GTGAGGATTAAGATCATTA 54
Megabat         --CTTTCTTGGGAAGCCAATTAGGCC-CCCAGTTGGCAGCATCAGGGATTAATATGATTA 57
Wallaby         TGCCTTCCAGGGAAGCCAATTAG----TGCTTGCCAACCTAGCTGG-ATTATTATGATTA 55
Oppossum        -GCCTTCTTGGGAAGCCAATTAA----TGCTTGCCAACCTAGCGGGGATTATTATGATTA 55
X.laevis        --CGTTTAAGGGAAGCCAATTAACA---CTTTGCAATTTTAGCTTGGATTACAGTGATTA 55
X.trop          --CGTTTATGAGAAGCCAATTAACAC---TTTGCAACCTTAGCTTGGATTACAATGATTA 55
Platypus        --CTGCTCTGGGAAGCCAATTAGCAAGCGCTTGCAAACCCAGTGTGGATTATTACGATTA 58


Felis           ATAATGCCCCCAATCTTCCAGGTGCTGATTC----AGCCAGGAGCTTAGGGAG--GGGAG 109
Dolphin         ATAACGCCCCCAATCTCGCAGGTGCTGATTC----AGCCAGGAGCTTAGGGAG--GGGAG 109
Canis           ATAACGCCCCCAATCTCCCAGGTGCTGATTC----AGCCAGGAGCTTAGGGAG--GGGAG 109
Chim            TGAACACCCCCAATCTCCCAGATGCTGATTC----AGCCAGGAGCTTAGGAGG--GGGAG 111
Gorilla         TGAACACCCCCAATCTCCCAGATGCTGATTC----AGCCAGGAGCTTAGGAGG--GGGAG 111
Orangutan       TGAACACCCCCAATCTCCCAGATGCTGATTC----AGCCAGGAGCTTAGGAGG--GGGAG 111
Human           TGAACACCCCCAATCTCCCAGATGCTGATTC----AGCCAGGAGCTTAGGAGG--GGGAG 110
Macaque         TGAACACCCCCAATCTCCCAGATGCTGATTC----AGCCAGGAGCTTAGGAGG--GGGAG 110
Marmoset        TGAACACCCC-AATCTCTCAGATGCTGATTC----AGCCAGGAGCTTAGGAGG--GGGAG 110
Bushbaby        TGA-CACCCC-AATCTCCCAGATGCTGATTC----AGCCAAGAGCTTAGGGAG--GGGAG 110
Mouse           TGAT--------ATCTCGCGGATGCTGAATC----AGCCTCTGGCTTAG-GGA-GAGAAG 100
Rat             TGAT--------ATCTCCCGGATGCTGAATC----AGCCTCTGGCTTAG-AGA-GAGAAG 101
Tree            CGAGCTCCTG-GATCGCCCGGGTGCCGAGTC----AGCA--GGCCTTAG-GGA-TAGA-G 105
GuineaPig       TGCGCC-----GATCTCCCGCAGGCTGAGTC----AGCG--CAGCTTAG-GCC-CAGAAG 100
Kangara         TGAGCACCCC-GATCTCCCCGGTGCTGATTC----AGCCAGGGGCTTAGAGGG-GGGAAG 112
Hedgehog        CCCC-------AATCTGCGGGCTGCTGAGTC----AGGC--AGGTTTAG-----CAGGAG 94
Pika            TGATAACCCCCAAACTCCCCGGTGCTGACTC----AGCTGAGAGCTTAGGGCG--GGAAG 112
Rabbit          TGATCACCCCCAATCTCCCCGGTGCTGACTC----AACCGGGAGCTTAGGGGGAGGGAAG 113
Horse           ATAACGCCCCCAATCTCCCAGGTGCTGATTC----AGCCAGGAGCTTAGGGAG--GGGAG 111
Elephant        ATAACGCCCCCAATCTCCCAGGTGCTGATTC----AGCCCAGAGCTTAGGGAG--GGGAG 109
Pig             ATAACGCCCCCAATCTCCCAGGTGCTGATTC----AGCCCAGAGCTTAGGGAG--GGGAG 110
Bos             ATAACGCCCCCAATCTCCGAGGTGCTGATTC----AGCCGGGAGCTTAGGGAG--GGGAG 110
Alpaca          ATAATGCCCCCAATCGCCCAGGTGCTGATTC----AGCCAGCAGCTTAGGGAG--GGGAG 110
```

```
Tenre           ATATCGCCCCCAATCTCCCAGGTGCTGATTC----AGTCAGAAGCTTAGGGAG---GGAG 107
Megabat         ATAACGCCCCCAATCTCCCAGGTGCTGATTC----AGCCAGGAGCTTAGGGAG--GGGAG 111
Wallaby         ATTATGCCCTGAATCTTCTGGGTGCTGACTCCATAGAAGGAGGGCTTACCCAG--AGGGG 113
Oppossum        GTCATGCCCTGAATCTTCTGGGTGCTGACACTAGGGAAGGAGGGCTTACCCAT--AGGGG 113
X.laevis        ATAGTGCGCTAAATCCTTT-GTTGCTGACGCTGGGGGTTGCAAGCTTACTCCA----GGT 110
X.trop          TTAGTGCGCTAAATCCTTTGTT-GCTGACGCTGGGGGTTGGCAGCTTACTTCA----GGT 110
Platypus        ATAATGCCCCCGATCCCCTGTTTGCTGACACCAGCCCCTCCAGCTTAGCTGAA----GGT 114


Felis           GTCACTTTATAAGGGCCTGGGGGGG--------- 134
Dolphin         GTCACTTTATAAGGGCCTGGGGGGG--------- 134
Canis           GTCACTTTATAACGGCCTGGGGGGG--------- 134
Chim            GTCACTTTATAAGGGTCTGGGGG----------- 134
Gorilla         GTCACTTTATAAGGGTCTGGGGG----------- 134
Orangutan       GTCACTTTATAAGGGTCTGGGGG----------- 134
Human           GTCACTTTATAAGGGTCTGGGGGG---------- 134
Macaque         GTCACTTTATAAGGGTCTGGGGGG---------- 134
Marmoset        GTCACTTTATAAGGGTCTGTGGGG---------- 134
Bushbaby        GTCACTTTATAAGGCTCTGGGGGG---------- 134
Mouse           GTCACTTTATAAGGGTCTGGGGGGGGTCAGTGCC 134
Rat             GTCACTTTATAAGGGTCTGGGGGGGGTC------ 129
Tree            GTCACTTAATAAGGGCCTGGGGGGGGTGG----- 134
GuineaPig       GTCACTTCATAAGGGCCTGGGGGGGCCAGGGCCA 134
Kangara         GTCACTTTATAAGGGTCTGGGG------------ 134
Hedgehog        GTCGCTTCATAAGGCCCTGGGGGGGGTCCC---- 124
Pika            GTCACTTTATAAGGGTCCTGGG------------ 134
Rabbit          GTCACTTTATAAGGGTCTGGG------------- 134
Horse           GTCACTTTATAAGGCCCTGGGGG----------- 134
Elephant        GTCACTTTATAAGGGCTTGGGGGGG--------- 134
Pig             GTCACTTTATAATGGCCTGGGGGG---------- 134
Bos             GTCACTTCATAAGGGCCTGGGGGG---------- 134
Alpaca          GTCACTTCATAAGGGCCTGGGGGG---------- 134
Tenre           GTCA-TTTATAAGGGTCTGGCGTGGGGG------ 134
Megabat         GTCACTTTATAAGGGCCTGGGGG----------- 134
Wallaby         GTCACTTTATAAGGACCTCAA------------- 134
Oppossum        GTCACTTTATAAGGACCTCAA------------- 134
X.laevis        GGGACTTTAAAAGGACGAGGGGAC---------- 134
X.trop          GGGACTTTAAAAGGACCCGGGGAC---------- 134
Platypus        TTGCCTCATAAAGGCTGCAG-------------- 134
```

References


(1) http://www.ebi.ac.uk/Tools/clustalw2/

(2) http://weblogo.threeplusone.com/

(3) Crooks GE, Hon G, Chandonia JM, Brenner SE WebLogo: A sequence logo generator, *Genome Research*, 14:1188-1190, (2004)
