# Supplementary material for: Cooperative activation of Xenopus rhodopsin transcription by paired-like transcription factors
Source: BMC Mol Biol. 2014 Feb 6;15:4. doi: 10.1186/1471-2199-15-4 (PMC3937059; doi:10.1186/1471-2199-15-4)
Supplement: Additional file 8 — Alignment of tetrapod rhodopsin proximal promoters. [file 1471-2199-15-4-S8.pdf]

Table 1 Sequences used in alignment of tetrapod rhodopsin promoters

Species	Ensembl Accession Number
Alpaca (*Vicugna pacos*)	ENSVPAG00000003334
Anole Lizard (*Anolis carolinensis*)	ENSACAG00000014258
Armadillo (*Dasypus novemcinctus*)	ENSDNOG00000015133
Bushbaby (*Otolemur garnettii*)	ENSOGAG00000010259
Cat (*Felis catus*)	ENSFCAG00000000092
Chicken (*Gallus gallus*	D00702 (GenBank)
Chimpanzee (*Pan troglodytes*)	ENSPTRG00000015379
Cow (*Bos taurus*)	ENSBTAG00000001310
Dog (*Canis familiaris*)	ENSCAFG00000004633
Dolphin (*Tursiops truncatus*)	ENSTTRG00000011273
Elephant (*Loxodonta africana*)	ENSLAFG00000001359
Rhodopsin 7 Fugu (*Takifugu rubripes*)	ENSTRUG00000004527
Gorilla (*Gorilla gorilla*)	ENSGGOG00000010728
Guinea Pig (*Cavia porcellus*)	ENSCPOG00000004984
Hedgehog (*Erinaceus europaeus*)	ENSEEUG00000008285
Horse (*Equus caballus*)	ENSECAG00000013709
Hyrax (*Procavia capensis*)	ENSPCAG00000013147
Kangaroo rat (*Dipodomys ordii*)	ENSDORG00000000911
Lesser hedgehog tenrec (*Echinops telfairi*)	ENSETEG00000000282
Macaque (*Macaca mulatta*)	ENSMMUG00000012734
Marmoset (*Callithrix jacchus*)	ENSCJAG00000017195
Medaka (*Oryzias latipes*)	ENSORLG00000010599
Megabat (*Pteropus vampyrus*)	ENSPVAG00000007112
Microbat (*Myotis lucifugus*)	ENSMLUG00000014786
Mouse (*Mus musculus*)	ENSMUSG00000030324
Mouse Lemur (*Microcebus murinus*)	ENSMICG00000008063
Opossum (*Monodelphis domestica*)	ENSMODG00000006488
Orangutan (*Pongo pygmaeus*)	ENSPPYG00000013415
Pig (*Sus scrofa*)	ENSSSCG00000011590
Pika (*Ochotona princeps*)	ENSOPRG00000011547
Platypus (*Ornithorhynchus anatinus*)	ENSOANG00000003778
Rabbit (*Oryctolagus cuniculus*)	ENSOCUG00000013036
Rat (*Rattus norvegicus*)	ENSRNOG00000011144
Squirrel (*Spermophilus tridecemlineatus*)	ENSSTOG00000004402
Tetraodon (*Tetraodon nigroviridis*)	ENSTNIG00000017909
Tree Shrew (*Tupaia belangeri*)	ENSTBEG00000011354
Turkey (*Meleagris gallopavo*)	ENSMGAG00000010301
Wallaby (*Macropus eugenii*)	ENSMEUG00000007196
