# Supplementary material for: Cooperative activation of Xenopus rhodopsin transcription by paired-like transcription factors
Source: BMC Mol Biol. 2014 Feb 6;15:4. doi: 10.1186/1471-2199-15-4 (PMC3937059; doi:10.1186/1471-2199-15-4)
Supplement: Additional file 9: Table S2 — List of primers used in this study. [file 1471-2199-15-4-S9.pdf]

PCR product/construct	sense primer (5'-3')	antisense primer (5'-3')
Histone H4	CGGGATAACATTCAGGGTATCACT	ATCCATGGCGGTAACTGTCTTCCT
Rax1a	CATATGAATACGGCTCAGGGC	AAATAAGGGTTTCGCCTGGC
Rax1b	GGGAGGACATTCTAACAAATCAGTGGAC	CCCTGACCTAGAGAGAGTCACAGAGACC
Rax2a	AGCTTTTACGAAACGCTATACCTCAAC	AATACTGATTTACCAGACCCAATGAAG
Rax2b	GGCAGCCTATCTGATGAAAACAGC	CTGACCCAGTGAAGACACAGTGAAG
pCS2-Rax1a	GAATTCAACACCTCAAGGGTCCTCAATG	CTCGAGCATATGTTTTACCAAGGCTTGCC
pCS2-Rax1b	GAATTCTCAATGCACCTGCACAGTCCTCC	CTCGAGCTGTGCCGTATTCATATGGTTTGCC
pCS2-Rax2a	GAATTCGCAGCTAAGTGCAGTTCAGG	CTCGAGTCAGATTGGCTGCCATG
pCS2-Rax2b	GAATTCAACAAGAACCAGCGTACCCAAG	CTCGAGATCAGATAGGCTGCCATGTTTTATC
Rax1a	GAGGCCATCTTGGGGTTTGTGAA	GGATGAATCTCCTCGGTCATTTTGTG
Rax1b	GAGGCCATCTTAGGGTTTGCCAA	GATGAATCTCCTCCGTCATTTTGTGG
Rax2a	AATACTGATTTACCAGACCCAATGAAG	AGCTTTTACGAAACGCTATACCTCAAC
Rax2b	GGCAGCCTATCTGATGAAAACAGC	CTGACCCAGTGAAGACACAGTGAAG
ΔRetBat1	AACTGCAGTAGTGCGCTAAATCCTTTG	AACTGCAGCCCTTAAACGTATTAAGTCTACAC
m1, m5, m7, m9, m10	CCAAAAGCTTTAAGCCAAGGAACACTCTATT	TTGGAATAGAGTGTTCCTTGGCTTAAAGCTT
m2, m5	CCAAAAGCTTTTGGAGGACAGTGCTATT	TTGGAATAGCACTGTCCTCCAAAAGCTT
m3	GCAATTTTAGCTTGGATTACAGTGAGGAATAGTGCGCTAAATCCTTTGTTG	CAACAAAGGATTTAGCGCACTATTCCTCACTGTAATCCAAGCTAAAATTGC
m4, m7, m9, m10	CCAAAAGCTTTCAAGCGGACTCCAGGCTATT	TTGGAATAGCCTGGAGTCCGCTTGAAAGCTT
m6, m8, m9, m10	CCAAAAGCTTTTGGAGGACAGTGAGGAACTATT	TTGGAATAGTTCCTCACTGTCCTCCAAAAGCTT
503 UP	CCCGGGCCACCACAGATGTCAAACGGACTG	ACGCGTGAGCTGCTACCATGAACGGAACAGA
503 UP	GCTAGCGGCCATGCGGTCCTAACGTCT	AGATCTGATCCCTAGAAGCCTGTGATCCCAAC
FRT-Hygromycin construct	GGATCCGATTAGGGTGATGGTTCACGTACCTAGAAGT	GTCGACTTTACACTTTATGCTTCCGGCTCGTATGT
